# Suppression of Estrogen Receptor Alpha Inhibits Cell Proliferation, Differentiation and Enhances the Chemosensitivity of P53-Positive U2OS Osteosarcoma Cell

**DOI:** 10.3390/ijms222011238

**Published:** 2021-10-18

**Authors:** Jir-You Wang, Chao-Ming Chen, Cheng-Fong Chen, Po-Kuei Wu, Wei-Ming Chen

**Affiliations:** 1 Department of Orthopaedics, Taipei Veterans General Hospital, Taipei City 112, Taiwan; yollywang@gmail.com (J.-Y.W.); drcmchen8@gmail.com (C.-M.C.); cf_chen@vghtpe.gov.tw (C.-F.C.); wmchen@vghtpe.gov.tw (W.-M.C.); 2 Department of Orthopaedics, Therapeutical and Research Center of Musculoskeletal Tumor, Taipei Veterans General Hospital, Taipei City 112, Taiwan; 3 Institute of Traditional Medicine, School of Medicine, National Yang Ming Chiao Tung University, Taipei 112, Taiwan; 4 Institute of Clinical Medicine, School of Medicine, National Yang Ming Chiao Tung University, Taipei 112, Taiwan; 5 School of Medicine, National Yang Ming Chiao Tung University, Taipei 112, Taiwan

**Keywords:** osteosarcoma, estrogen receptors, chemotherapy

## Abstract

Osteosarcoma is a highly malignant musculoskeletal tumor that is commonly noticed in adolescent children, young children, and elderly adults. Due to advances in surgery, chemotherapy and imaging technology, survival rates have improved to 70–80%, but chemical treatments do not enhance patient survival; in addition, the survival rate after chemical treatments is still low. The most obvious clinical feature of osteosarcoma is new bone formation, which is called “sun burst”. Estrogen receptor alpha (ERα) is an essential feature of osteogenesis and regulates cell growth in various tumors, including osteosarcoma. In this study, we sought to investigate the role of ERα in osteosarcoma and to determine if ERα can be used as a target to facilitate the chemosensitivity of osteosarcoma to current treatments. The growth rate of each cell clone was assayed by MTT and trypan blue cell counting, and cell cycle analysis was conducted by flow cytometry. Osteogenic differentiation was induced by osteogenic induction medium and quantified by ARS staining. The effects of ERα on the chemoresponse of OS cells treated with doxorubicin were evaluated by colony formation assay. Mechanistic studies were conducted by examining the levels of proteins by Western blot. The role of ERα on OS prognosis was investigated by an immunohistochemical analysis of OS tissue array. The results showed an impaired growth rate and a decreased osteogenesis ability in the ERα-silenced P53(+) OS cell line U2OS, but not in P53(−) SAOS2 cells, compared with the parental cell line. Cotreatment with tamoxifen, an estrogen receptor inhibitor, increased the sensitivity to doxorubicin, which decreased the colony formation of P53(+) U2OS cells. Cell cycle arrest in the S phase was observed in P53(+) U2OS cells cotreated with low doses of doxorubicin and tamoxifen, while increased levels of apoptosis factors indicated cell death. Moreover, patients with ER−/P53(+) U2OS showed better chemoresponse rates (necrosis rate > 90%) and impaired tumor sizes, which were compatible with the findings of basic research. Taken together, ERα may be a potential target of the current treatments for osteosarcoma that can control tumor growth and improve chemosensitivity. In addition, the expression of ERα in osteosarcoma can be a prognostic factor to predict the response to chemotherapy.

## 1. Introduction

Osteosarcoma (OS) is the most common sporadic malignant tumor that occurs in childhood or adolescence and is frequently observed in parts of the body characterized by rapid bone growth, such as the knee joint, distal femur, proximal tibia, and proximal humerus [1]. The clinical manifestations of OS include new bone formation and tumor and periosteal reactions, such as sunburst features or onion skin, that are similar to those of osteoprogenitors [2]. The most common subtypes include the osteoblastic, fibroblastic, and chondroblastic types according to imaging diagnosis [3]. Although molecular markers for osteosarcoma diagnosis are still lacking, some genetic studies have demonstrated that mutations in tumor suppressor genes (TSGs), such as P53, Rb, and c-Myc, in osteosarcoma may be related to therapy efficiency and prognosis [4,5,6,7,8,9,10]. Mutations in these genes have been reported in established animal or cell models of osteosarcoma [4,8,11], indicating the role of these genes in the occurrence of osteosarcoma. Recent evidence shows that mesenchymal stem cells (MSCs) may be the progenitor cells that form OS due to certain genetic mutations [12,13,14,15,16]. An imbalance between the proliferation and differentiation of MSCs has been demonstrated to be associated with tumorigenesis in many cancers, including OS [17,18]. The factors that contribute to osteogenesis in OS are similar to those observed in MSCs [12,16,18], and undifferentiated stem cells that exhibit uncontrolled proliferation cause OS tumorigenesis [18]. In addition, defects in the differentiation of osteoprogenitors are postulated to be responsible for OS tumorigenesis or malignant changes and are considered potential therapeutic targets of the current chemotherapy regimens [18].

Doxorubicin is a member of the family of anthracycline drugs commonly used in the treatment of many cancers [19], including osteosarcoma [20]. The mechanisms of the cytotoxic effect of doxorubicin have been postulated to involve G2/M arrest [21] and G1/S arrest or Fas-mediated apoptosis [22]. Despite the efficient therapeutic responses to doxorubicin, there have been increasing reports that indicate that increasing the dosage leads to more severe side effects [23,24], therapy relapse [25], and drug resistance [26]. In osteosarcoma, the nonresponse rate to chemotherapy is approximately 40–50% [27], and the non-effectiveness of chemotherapy leads to poor prognosis and a lower survival rate. Enhanced efficiency or increased sensitivity of cancer cells to chemotherapy will be very important for improving tumor therapy.

New bone formation is a common feature of various kinds of bone tumors. The osteogenesis process is strictly controlled by various factors, such as transforming growth factor beta (TGF-β), bone morphogenic proteins (BMPs), runt-related gene-2 (RUNX2), and the downstream factors of these three major pathways [28,29]. Recently, steroid hormones have been widely due to their critical role in controlling bone formation. The loss of estrogen or the functional deficiency of the estrogen receptor (ER) suppresses osteoblast growth and impairs osteogenesis [30]. The activation of the ER, especially ER-alpha (ERα), triggers the downstream Wnt//beta-catenin signaling cascade that promotes osteogenesis [31]. Because of the critical role of ER in bone formation, whether the control of ER can modulate the new bone formation and affect the prognosis or chemosensitivity of bone tumors is an interesting issue for further study.

Several lines of evidence demonstrate that ER is a potential target for the treatment of OS. For example, estrogen and selective estrogen receptor modulators (SERMs) protect ER-expressing OS cells from apoptosis through the activation of the interleukin 6 (IL-6)-related pathway [32]. ERβ maintains the cell viability and promotes the cell migration of OS cells through the PI3K/Akt pathway [33]. A recent investigation suggested that targeting ERα-sensitive OS treated with methotrexate [34] enhances the cytotoxic effects on OS when combined with doxorubicin treatment [35]. However, many studies have shown that the *P53* tumor suppressor gene plays important roles in affecting the prognosis of OS patients [36,37]. Nevertheless, the crosstalk between ERα and P53 in OS chemoinsensitivity remains unknown. Therefore, the aim of this study was to investigate the role of ER in OS prognosis and to elucidate the combined effects of targeting ER with chemoadjuvants on different types (with or without P53 expression) of OS cells.

## 2. Results

### 2.1. ERα Positive Expression Pattern in OS Patients Was Correlated with Increased Tumor Size and ALP and LDH Levels

**Figure 1:**
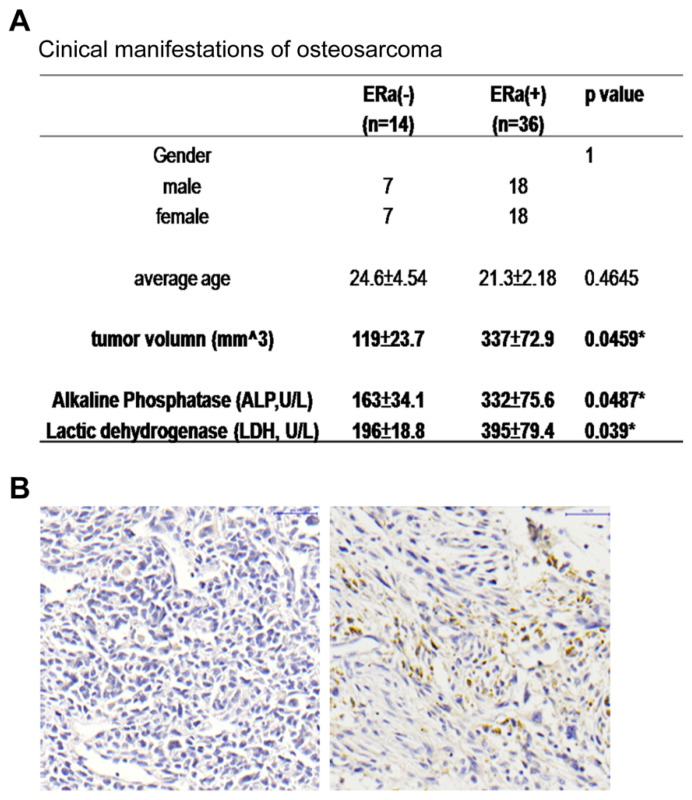
ERα positive expression pattern in OS patients was correlated with increased tumor sizes and ALP and LDH levels. (**A**) The enrolled patients’ information; ER(+) subjects showed larger tumor sizes and higher ALP and LDH levels. (**B**) The immunostaining of ERα on OS sections showed positive brown color. * *p* < 0.05.

The ERα expression level of the analyzed OS tumor sections was identified by immunostaining, and the tissue array sections were divided into two groups: ER(+) and ER(−) (Figure 1A). Among the 50 tissue spots from the primary OS patients, 36 spots (72%) were ER(+) and 14 spots (28%) were ER(−), and there was no significant difference in the age and gender of the patients in these two groups. In addition to the larger tumor size, increased alkaline phosphatase (ALP) and lactic dehydrogenase (LDH) were observed in the ER(+) patients (Figure 1B). Together, these data suggest that ER expression in OS is important for tumor development and size determination.

### 2.2. ERα Knock down Suppressed the Growth Rate of P53-Positive U2OS Cells but Not of P53-Negative SAOS2 Cells

**Figure 2:**
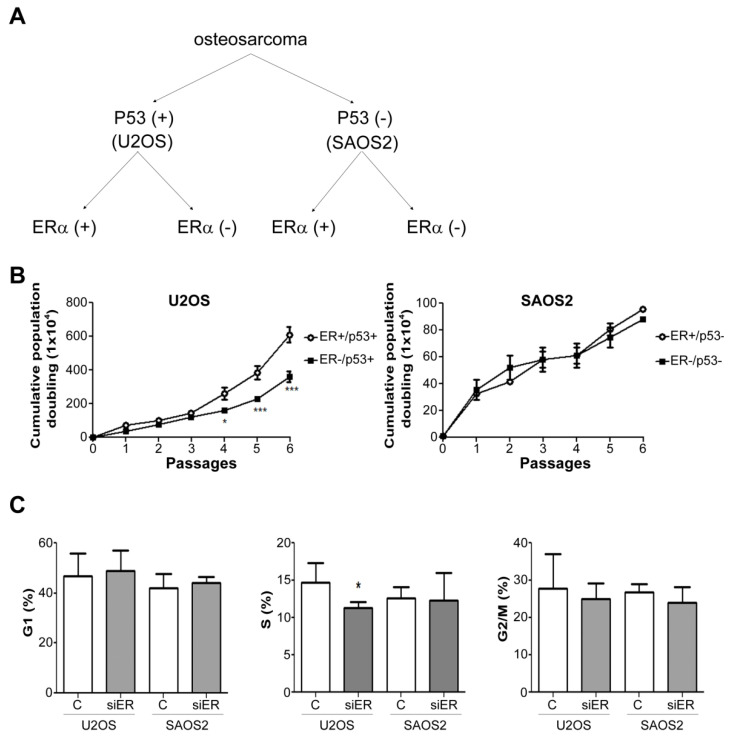
ERα knockdown suppressed the growth rate of the P53-positive U2OS cells but not the P53-negative SAOS cells. (**A**) Flow chart of the experimental design. Two types of OS cell lines were tested for the effects of ERα, including P53(+) U2OS cells and P53(−) SAOS2 cells. (**B**) The cells were continuously seeded in complete medium for 6 passages, and the cumulative population doublings were calculated by trypan blue assay. (**C**) The cell cycle of individual cells was analyzed by flow cytometry. * *p* < 0.05, and *** *p* < 0.005 compared to the parental cells in the individual passages.

Since P53 mutations were observed to affect the prognosis of some OS patients, we used two types of OS cell lines, namely, U2OS, which expresses normal P53 levels [P53(+)], and P53-mutated cells, SAOS2, which do not express P53 [P53(−)], to examine the role of ER in different types of OS (Figure 2A). During six continuous passages, ER knockout in the P53(+) cells obviously decreased the growth rate after the fourth passage (Figure 2B, left), while there was no significant difference in the P53(−) SAOS2 cells (Figure 2B, right). The cell cycle analysis by flow cytometry also indicated S phase decreased in the P53+/ER− U2OS cells (Figure 2C, middle).

### 2.3. Knock down of ERα Suppressed the Osteogenesis Ability in Both P53+ U2OS and P53− SAOS2Cells

**Figure 3:**
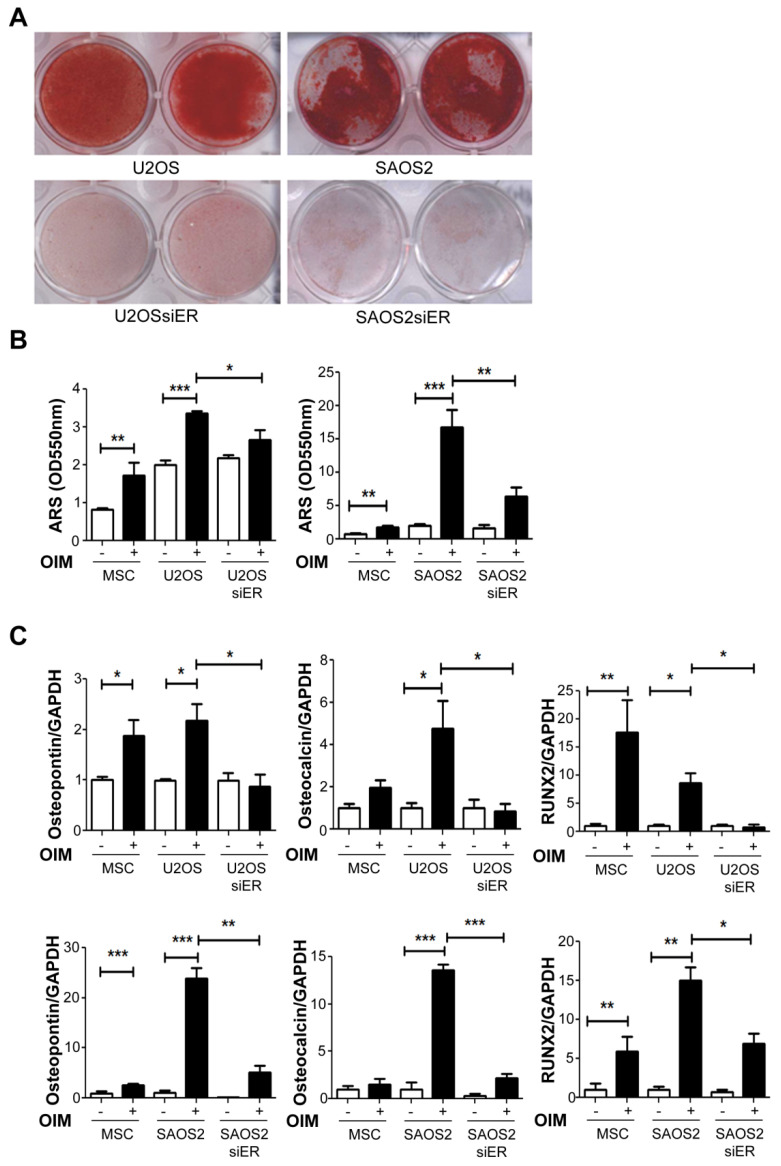
Knockdown of ERα suppressed the osteogenesis abilities of both the P53+ U2OS and P53− SAOS2 cells. (**A**) The cells were cultured in OIM for up to 2 weeks to induce osteogenesis and were analyzed by ARS staining. (**B**) ARS staining was conducted, and the OD values were measured for quantification. (**C**) The gene expression levels of osteopontin, osteocalcin, and RUNX2 were analyzed by quantitative RT-PCR assay7 days after induction. Anormal mesenchymal stem cell (MSC) line was used as a positive control. Compared to the parental cells, the SiERα cells had significantly decreased levels of osteogenic genes. * *p* < 0.05, ** *p* < 0.01, and *** *p* < 0.005 compared to parental cells.

ERα was reported to play a critical role in the osteogenesis process [38,39]. In our system, both U2OS (P53+) and SAOS2 (P53−) OS cell lines showed ARS staining that was highly positive after two weeks of incubation in osteogenic induction medium (Figure 3A, upper panel), indicating high osteogenic abilities. The knockdown of ERαobviously decreased the osteogenic abilities of both the OS cell lines (Figure 3A, lower panel) that be quantified by ARS staining (Figure 3B) The genes related to the osteogenesis process, such as osteopontin, osteocalcin, and RUNX2, were significantly decreased in the SiERα cells on P53 positive U2OS groups but not in P53 negative SAOS2 cells (Figure 3C), indicating that the knockdown of ERα impaired the expression levels of osteogenesis-related genes that suppressed the osteogenic abilities of the P53 positive U2OS cells.

### 2.4. Silencing of ERα in P53-Positive U2OS Cells Suppressed Colony Formation Ability after Combined Treatment with Doxorubicin

**Figure 4:**
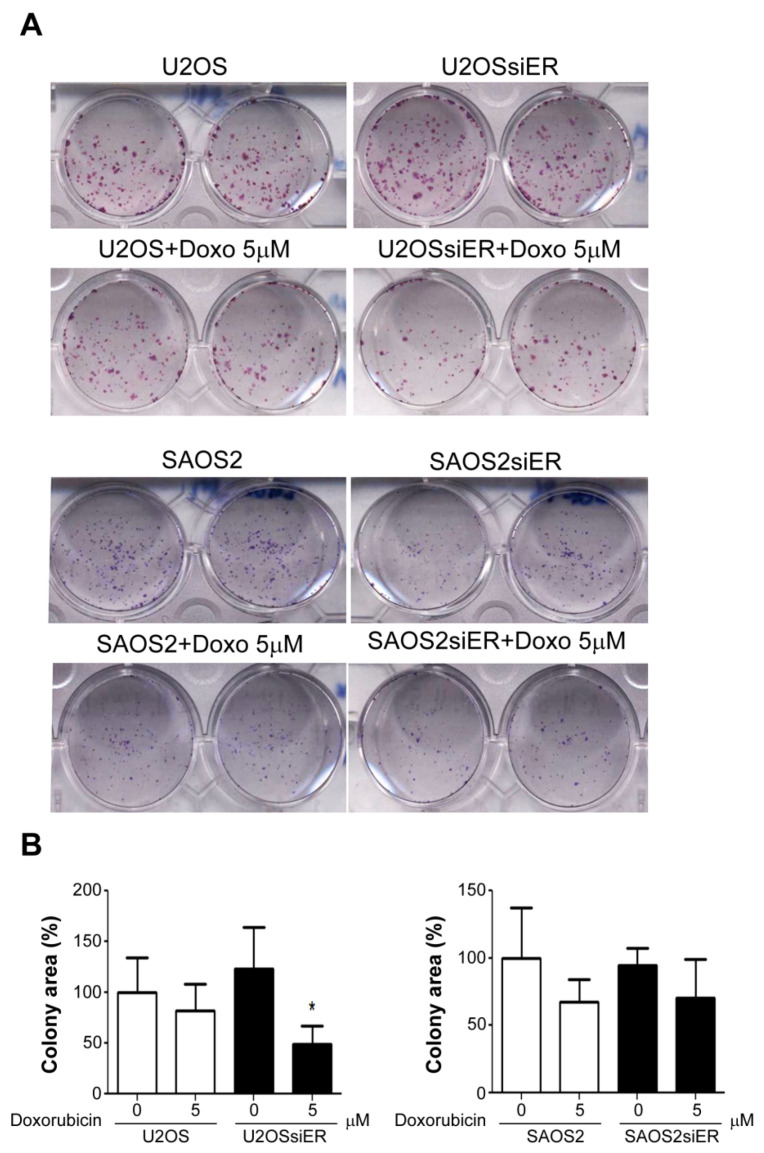
Silencing of ERαin P53-positive U2OS cells suppressed colony formation abilities after treatment with doxorubicin. (**A**) The cells were seeded at 1000 cells/well in a 6-well plate and incubated for 7 days, followed by crystal violet staining. Groups of more than 250 cells were stained blue. (**B**) The total colony area was quantified by ImageJ software, and * *p* < 0.05 was considered to be a significant difference.

A colony formation assay was performed to assess the tumorigenesis abilities of the tumor cells. In low-density culture, compared to wild-type ERα expression (normalized to 100%), the silencing of ERα showed no significant effects on the colony formation abilities of either P53+ (122.8 ± 23.66%) or P53− (94.4 ± 7.42%) OS cells. The silencing of ERα in the P53+ U2OS cells induced sensitivity to doxorubicin treatment that suppressed colony formation (48.9 ± 10.51%), but this effect was not observed in the P53− SAOS cells (70.3 ± 16.69%) (Figure 4A,B), indicating that targeting ERα enhanced the tumor suppression effect of doxorubicin.

### 2.5. Combined Treatment with Tamoxifen Enhanced the Growth Inhibition Effects of Doxorubicin on P53(+) U2OS Cell by Suppressing CDK2 and Cyclin A and Inducing Apoptosis

**Figure 5:**
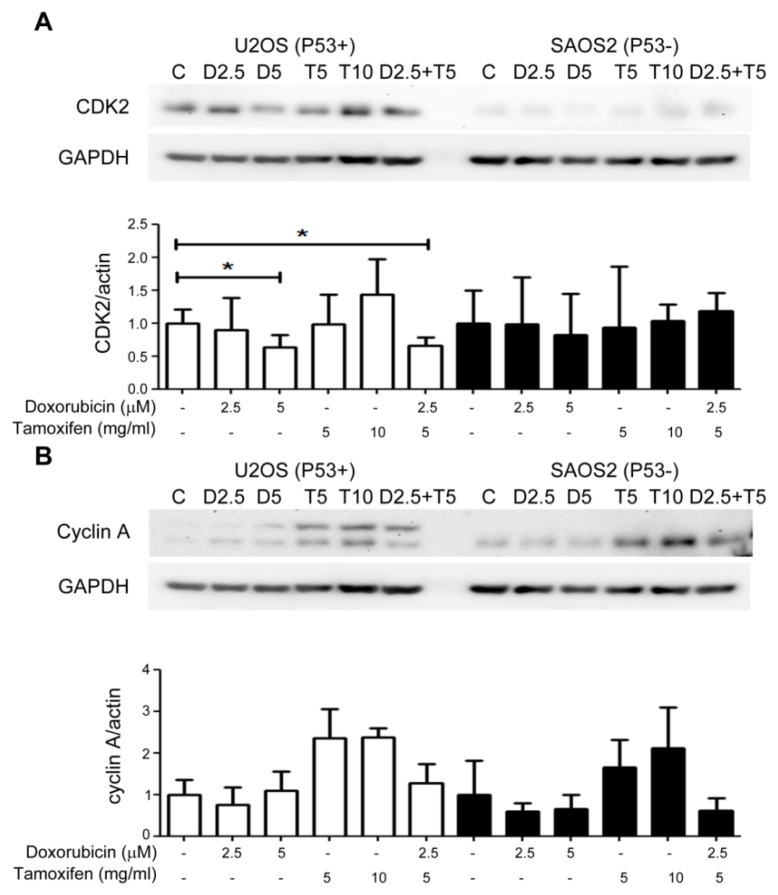

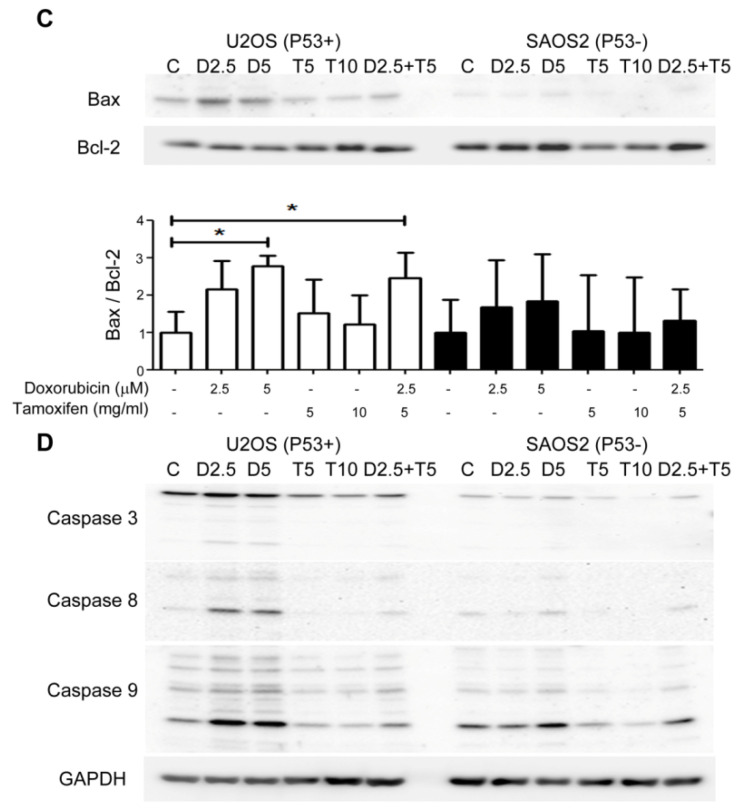
Combined treatment with tamoxifen enhanced the growth inhibition effects of doxorubicin on the P53(+) U2OS cell lines by suppressing CDK2 and cyclin A and inducing apoptosis. The expression levels of checkpoint factors for the S phase in the cell cycle, such as CDK2 and cyclin A, were examined by Western blot. (**A**) Treatment with doxorubicin obviously suppressed CDK2, and similar effects were achieved by the combination treatment with tamoxifen at a lower dose. (**B**) The effects of the combination treatment showed a trend of in habiting the expression of cyclin A, although there was no significant difference. (**C**) The ratio of a pro-apoptotic protein (Bax) and an anti-apoptotic protein (Bcl-2) in the combined treatment group was similar to that in the high-dose doxorubicin treatment group. (**D**) The programmed death factors, i.e., the caspases, were cleaved in the combination treatment group, indicating more efficient apoptosis. * *p* < 0.05 compared to the control group (C, no treatment). D2.5: doxorubicin 2.5 μM; D5: doxorubicin 5 μM; T5: tamoxifen 5 μg/mL; T10: tamoxifen 10 μg/mL; and D2.5+T5: doxorubicin 2.5 μM + tamoxifen 5 μg/mL.

Treatment of the OS cell lines with increasing doses of doxorubicin suppressed cell growth by inhibiting the expression of cyclin A and CDK2, while no suppression effects were observed when the cells were treated with tamoxifen (Figure 5A). The efficiency of this suppression achieved by a low dose of doxorubicin (2.5 μM) combined with a low dose of tamoxifen (5 μg/mL) was similar to that achieved by a high dose of doxorubicin (5 μM) (Figure 5A,B). In addition to the suppression of the cell cycle, apoptosis proteins were induced in the combination treatment groups, and this induction was more significant in the P53(+) U2OS cells (Figure 5C,D).

### 2.6. ERα Low Expression Patterns in P53-Positive OS Patients Were Associated with Better Responses to Chemotherapy and Smaller Tumor Sizes

Since the role of ERαdiffered in the P53(+) or P53(−) OS cell lines, we next analyzed patient outcomes in these two groups. From all the 50 OS tumor sections, the most common expression pattern was the ER(+)/P53(+) pattern (Table 1, 29/50 cases). In terms of the chemoresponsive rate, ER(−)/P53(+) patients showed a significantly good response (necrosis rate > 90%) compared to the other three groups. In terms of tumor size, P53(+) or P53(−) OS sections that were ER(−) seemed to be smaller than the P53(+) or P53(−) OS sections that were ER(+). The lung metastasis rate and 5-year survival rate were not obviously different between these phenotypes (Table 1).

## 3. Discussion

Regarding osteosarcoma therapy, the control of tumor size before surgery and prevention of metastasis are key goals that improve survival. Current chemo-adjuvants used to treat osteosarcoma before surgery, including doxorubicin, cisplatin, ifosfamide, and methotrexate [40], improve the survival rate by up to 70%. However, since approximately 40% of patients remain chemo-nonresponsive [27], chemoefficiency must still be improved. The challenge of osteosarcoma therapy is the unclear specific markers for diagnosis and treatment. There are many genetic mutations observed in osteosarcoma patients. Furthermore, the mutation of P53 has been reported to regulate the onsetof osteosarcoma [41] and in high grade osteosarcoma patients, almost 38% presented the mutation form of P53 while patients suffering from Li Fraumeni syndrome with a P53 congenital mutation have a risk of developing osteosarcoma that is restricted to 12% [6,42]. In our previous unpublished data of NGS sequencing of tumor specimens, P53 mutation can be as high as >50%. The removal of P53 and Rbin stem cells was reported to increase the development of osteosarcoma in a mouse model [43]. From our previous study, the mutation of P53 cannot induce the occurrence of osteosarcoma on human stem cells, which indicated the different roles of P53 in species [4]. Furthermore, mutations in P53 which abolish its function, which are most commonly observed in osteosarcoma, have been postulated to confer resistance to many chemotherapeutic agents, including doxorubicin [44]. In this study, the suppression of ERα enhanced the chemosensitivity on P53-positive U2OS cells, which implied that combined treatment of ER-targeting medicine may support the current treatment on P53 normal expression osteosarcoma patients but not P53 mutation groups.

The estrogen/estrogen receptor axis is well known to play an essential role in modulating osteoblast maturation and improving osteoblast activity [45,46,47]. Nonetheless, the role of estrogen or estrogen receptor in osteosarcoma remains controversial. For example, treatment of the osteosarcoma cell line U2OSwith 17β-estradiol promotesproliferation, colony formation, and migration through an ERα-dependent pathway [48], while treatment of the other osteosarcoma cell line MG63 suppresses cell proliferation and migration through an ERα-independent pathway [49]. Ruza et al. [50] detected estrogen receptor expression in 58 osteosarcoma patients without any variants and showed that estrogen receptor expression is a critical risk factor in osteosarcoma [51]. However, in recent research, Lillo et al. [52] demonstrated that no estrogen receptor was detected in 11 osteosarcoma patients and Dohi et al. did not detect the expression of ERαin28 osteosarcoma patients [53]. In our study, from the results of the immunohistochemical analysis of the osteosarcoma tissue array, 36 patient sections among the 50 samples were positive for ERα, and exhibited significantly larger tumor sizes (Figure 1A). Thus, the expression of ERα was not found in all osteosarcoma patients and the existence of ERα may provide a therapeutic target for these patients.

To identify the effects of combination therapies on osteosarcoma in vitro, two types of osteosarcoma cell lines were studied, including P53(+) U2OS cells and P53(−) SAOS2 cells, to mimic the different osteosarcoma types [54]. Of these two cell lines, the SAOS2 cells expressed high levels of ERα [55], while the expression of ERαwas not stable inU2OS cells [48,52,56,57]. According to Osuna et al. in 2019 [52], the downregulated expression of ERα in osteosarcoma patients may be due to promoter methylation and in osteosarcoma cell lines, the expression of ERα cannot be detected in three osteosarcoma cell lines, including 143B, MG63, and U2OS by Western blot using MCF7 breast cancer cell line as positive control. However, from the PCR data, the nonmethylated ESR1 can still be observed in U2OS and MG63 cells. From our results, the expression level of ERαcan be detected in both SAOS2 and U2OS cell lines, and the knockdown efficiency by ERαshRNAs was approximately 60% (Supplementary Figure S1). Thus, the Western blot data from Osuna may be due to the high expression of ERα on MCF7 and the exposure time for other three osteosarcoma cell lines may not enough to detect the lower level of ERα compared to MCF7. The knockdown of ERα showed stronger effects on cell proliferation, colony formation, and chemosensitivity in the P53(+) U2OS cells than in the P53(−) cells and similar effects on osteogenesis differentiation in both the P53(+) and P53(−) cells. Based on the clinical outcome, ERα-negative P53(+) patients showed a better chemoresponse (necrosis rate > 90%, Table 1), indicating that ERα may be a potential target for treatments with combinations of current chemoadjuvants to improve the chemosensitivity of osteosarcoma therapy.

In summary, the suppression of ER enhanced the chemoresponse of P53(+) U2OS tumors, but not P53(−) SAOS tumor cells, and could be a future therapeutic target for chemotherapy agents. The expression of ERα is different in osteosarcoma patients; from our data, almost half of the patients were ERα positive, which indicated the potential of ERα-targeted therapy to support the current chemotherapy on P53-positive osteosarcoma.

## 4. Materials and Methods

### 4.1. Cell Lines and Culture Conditions

Human osteosarcoma cell lines were purchased from American Tissue Culture Collection (ATCC, Rockville, Gaithersburg, MD, USA) and cultured in high-glucose Dulbeccos modified Eagle medium (HG-DMEM, GIBCO-BRL, Gaithersburg, MD, USA) with 10% FBS (GIBCO-BRL, Gaithersburg, MD, USA). Two cell lines, U2OS (HTB-96), which was reported as a P53 wild-type cell line [58], and SAOS2 (HTB-85), which does not express P53 [59], were maintained in HG-DMEM and subcultured by 0.25% trypsin (GIBCO-BRL, Gaithersburg, MD, USA) digestion.

### 4.2. Transfection and Lentiviral-Mediated Transduction

The expression plasmids and lentiviral particles expressing short hairpin RNA targeting ERα (ESR1, TRCN0000003300, TRCN0000338156) were provided by the National Science Council RNAi core facility at Academia Sinica Taiwan. Once the cells reached confluence, they were infected by lentivirus with 8 μg/mL polybrene (Sigma, St. Louis, MO, USA). Twenty-four hours post infection, the medium was replaced with fresh growth medium containing puromycin (3 μg/mL) to select the stable clones. The maintenance dose of puromycin in the growth medium was 0.3 μg/mL.

### 4.3. Calculation of Cell Growth

Cells were initially seeded at 5 × 10^3^ cells/well in 96-well plates and incubated in complete medium for three days. Then, the cell growth rate was determined by an MTT (tetrazolium-based colorimetric assay, MTT) cell proliferation assay kit (Sigma, St. Louis, MO, USA). The cell numbers were evaluated each day by incubation of the cells with the MTT dye (5 mg/mL) at 37 °C for 4 h followed by solubilization of the dye with DMSO. The absorbance was measured at 570 nm by a multiscanautoreader (TECAN, M1000 PRO). The results obtained each day were compared to those obtained on day 0 (*n* = 3), and the results are presented as cumulative population doublings ± SD.

### 4.4. Flow Cytometry for Cell Cycle Analysis

The cell cycle progression of each cell line was analyzed by flow cytometry assay. In brief, suspensions of the cells were fixed with 75% ice-cold ethanol and then incubated with propidium iodide (5 μg/mL PI in 0.1% Triton X-100, Sigma, St. Louis, MO, USA) for 20 min. The cells were analyzed by a FAC Scan flow cytometer running Cell Quest software (Becton Dickinson, San Jose, CA, USA).

### 4.5. Colony Formation Assay

Cells transfected with or without ESR1 siRNA were seeded at a density of 1000 cells/well in 6-well plates. The colonies were counted 14 days later after fixation with 3.7% methanol and staining with 0.1% crystal violet. Groups of more than 50 cells were scored as a colony. Each treatment was performed in triplicate [60].

### 4.6. Osteogenesis Induction and Alizarin Red S Staining

Osteogenesis was induced by osteogenic induction medium (OIM: complete growth medium containing 50 mg/mL ascorbate-2 phosphate (Sigma, St. Louis, MO, USA), 10^−8^ M dexamethasone (Sigma, St. Louis, MO, USA), and 10 mM β-glycerophosphate (Sigma, St. Louis, MO, USA)) for the in vitro differentiation into osteoblasts. Confluent cells seeded in 12-well plates were cultured in OIM for 2 weeks. After fixation with cold ethanol for 2 h, the cells were incubated with Alizarin Red S (ARS; Sigma, St. Louis, MO, USA) for 20 min and were analyzed byOD550 measurement. All the groups were compared to the cells treated without OIM as a control.

### 4.7. Immunohistochemistry on Tumor Tissue Array

Under the approval of the Institutional Review Board of Taipei Veterans General Hospital (2015-06-005AC), OS tumor samples were collected from the Department of Orthopaedics and Traumatology, Taipei Veterans General Hospital, from 1992–2011. The cohort study was followed to determine mortality until 2014. The tumor size, and the levels of ALP and LDH were collected from medical record under routine inspection. The tissue arrays collected from 57 OS patients were analyzed by immunohistochemistry for the expression of ERα and P53. Missing samples, patients who were lost to follow-up, and duplicate collections were excluded, and a total of 50 tumor spots from each subject were analyzed in this assay. The dewaxed and rehydrated tissue array sections were retrieved by 10 mM sodium citrate buffer (pH 6.0) with 0.05% Tween 20 (Sigma, St. Louis, MO, USA) in a 95 °C water bath for 20 min and blocked by 3% H_2_O_2_ (Sigma, St. Louis, MO, USA). The antibodies against human ERα (mouse IgG anti-human, 1:100; GeneTex, Irvine, CA, USA) and P53 (mouse IgG anti-human, 1:100; Cell Signaling) were applied to the specimens. The DAKO LSAB kit (Dako Cytomation, Santa Clara, CA, USA) was used for detection. The expression levels of ERα and P53 in the tissue array sections were identified by more than two double-blinded researchers.

### 4.8. Western Blotting

Protein was extracted from the cell pellets by M-PER (Pierce, Rockford, IL, USA) containing a protease and phosphatase inhibitor cocktail (Halt™; Pierce, Rockford, IL, USA), and the protein concentration was determined by a BCA assay (Pierce, Rockford, IL, USA). The protein lysates were separated on SDS polyacrylamide gels, transferred to PVDF membrane filters and blocked with 5% non-fat milk in TBST (20 mM Tris-HCl [pH 7.6], 137 mM NaCl, 1% Tween 20). The membranes were then probed with primary antibodies overnight followed by the corresponding secondary antibodies and detected by chemiluminescence assay (Millipore, Bedford, MA, USA). The membranes were then exposed and scanned to measure the intensity of each band by using ImageMaster 2D Platinum version 5.0 (GE Healthcare Amersham Bioscience, Chicago, IL, USA). The primary antibodies included anti-ERα anti-CDK4, anti-cyclin D1, anti-CDK2, anti-cyclin A, and anti-cyclin B1 (1:500, GeneTex, Irvine, CA, USA), anti-Bax and anti-Bcl2 (1:500, Cell Signaling) and anti-GAPDH (1:1000; GeneTex, Irvine, CA, USA) as the internal control. The secondary antibodies included horseradish peroxidase-conjugated anti-rabbit or anti-mouse antibodies (1:2000; GeneTex, Irvine, CA, USA). The quantification of each protein expression level was normalized to that of the internal control GAPDH, and the expression levels of the parental lines U2OS or SAOS2 were referred to as 1.

### 4.9. Statistics

The data are presented as the mean ± standard error mean (SEM). Independent *t* tests were used to compare two independent samples, and one-way ANOVA with Bonferroni post hoc test was used to identify the significant differences among more than two groups. Chi-square (X^2^) was used to identify the significant differences between the expected frequencies and the observed frequencies. A *p*-value < 0.05 was considered statistically significant, and the *p*-values were labeled as follows: * *p* < 0.05, ** *p* < 0.01, and *** *p* < 0.005.

## Figures and Tables

**Table 1:** Clinical outcome of osteosarcoma comparing the ERα and P53 pattern.

	ERα(+)/P53(+)	ERα(−)/P53(+)	ERα(+)/P53(−)	ERα(−)/P53(−)	*p*-Value
	(n = 29)	(n = 11)	(n = 7)	(n = 3)	
Gender					0.6961
Male	16	7	2	0	
Female	17	6	1	1	
Average age	21.5 ± 2.3	25.3 ± 4.8	19.3 ± 4.9	13.5 ± 0.5	0.6874
Lung metastasis					0.7016
No metastasis	10	6	3	1	
Metastasis	19	5	4	2	
Chemoresponse rate					0.0358 *
Good response (necrosis rate > 90%)	13	8	1	0	
Poor response (necrosis rate < 90%)	16	3	6	3	
Tumor volume (mm^3^)	315 ± 84.7	113 ± 30.3	433 ± 63.9	140 ± 23.2	0.0151 *
5-year survival	21 (72.4%)	9 (81.8%)	3 (42.8%)	0 (0%)	0.0028 *

* *p* < 0.05.

## Data Availability

Data is contained within the article and Supplementary Materials.

## Supplementary Materials

The following are available online at https://www.mdpi.com/article/10.3390/ijms222011238/s1.
