# Supplementary material for: Suppression of Estrogen Receptor Alpha Inhibits Cell Proliferation, Differentiation and Enhances the Chemosensitivity of P53-Positive U2OS Osteosarcoma Cell
Source: Int J Mol Sci. 2021 Oct 18;22(20):11238. doi: 10.3390/ijms222011238 (PMC8540067; doi:10.3390/ijms222011238)
Supplement: Supplementary file 1 [file ijms-22-11238-s001.zip › Figure legends of supplement figures.pdf]

Figure legends of supplement figures

Supplement figure S1. Knockdown of estrogen receptor alpha on osteosarcoma cell lines, U2OS and SAOS2, by shRNA lentiviral vectors. The suppression of ERα expression on U2OS and SAOS2 cell lines were silenced by ERα lentiviral vectors with two different sequences (shESR1, TRCN0000003300, TRCN0000338156). From Western blot assay, plasmid of TRCN0000338156 was more efficient and the following experiments were assayed by this stable clone. The expression of p53 was undetectable on SAOS2 cell lines.

Supplement figure S2. Knockdown of estrogen receptor alpha on the other p53 negative MG63 osteosarcoma cell line showed no significant difference on cell growth and colony formation but suppressed osteogenesis ability. To test the effects of ERα on osteosarcoma cells, one more p53 negative cell line, MG63, was used to confirm the role of ERα. Similar to p53- SAOS2 cell, knockdown of ERα on MG63 also showed no significant different on cell growth (A) or colony formation (B). However, the osteogenesis ability was suppressed by ERα knockdown (C).

Supplement figure S3. The four expression patterns of ERα and p53 on osteosarcoma patient tissues. The expression pattern of ERα and p53 were assayed by immunohistochemistry on human osteosarcoma tissue arrays. Regards of the missing spot or replicated collections, 50 tissue sections were stained by anti-ERα and anti-p53 antibodies. There were four patterns on these tumor sections: ER+/p53+, ER-/p53+, ER+/p53-, and ER-/p53- groups.
