# Supplementary figures and images for: Suppression of Estrogen Receptor Alpha Inhibits Cell Proliferation, Differentiation and Enhances the Chemosensitivity of P53-Positive U2OS Osteosarcoma Cell
Source: Int J Mol Sci. 2021 Oct 18;22(20):11238. doi: 10.3390/ijms222011238 (PMC8540067; doi:10.3390/ijms222011238)

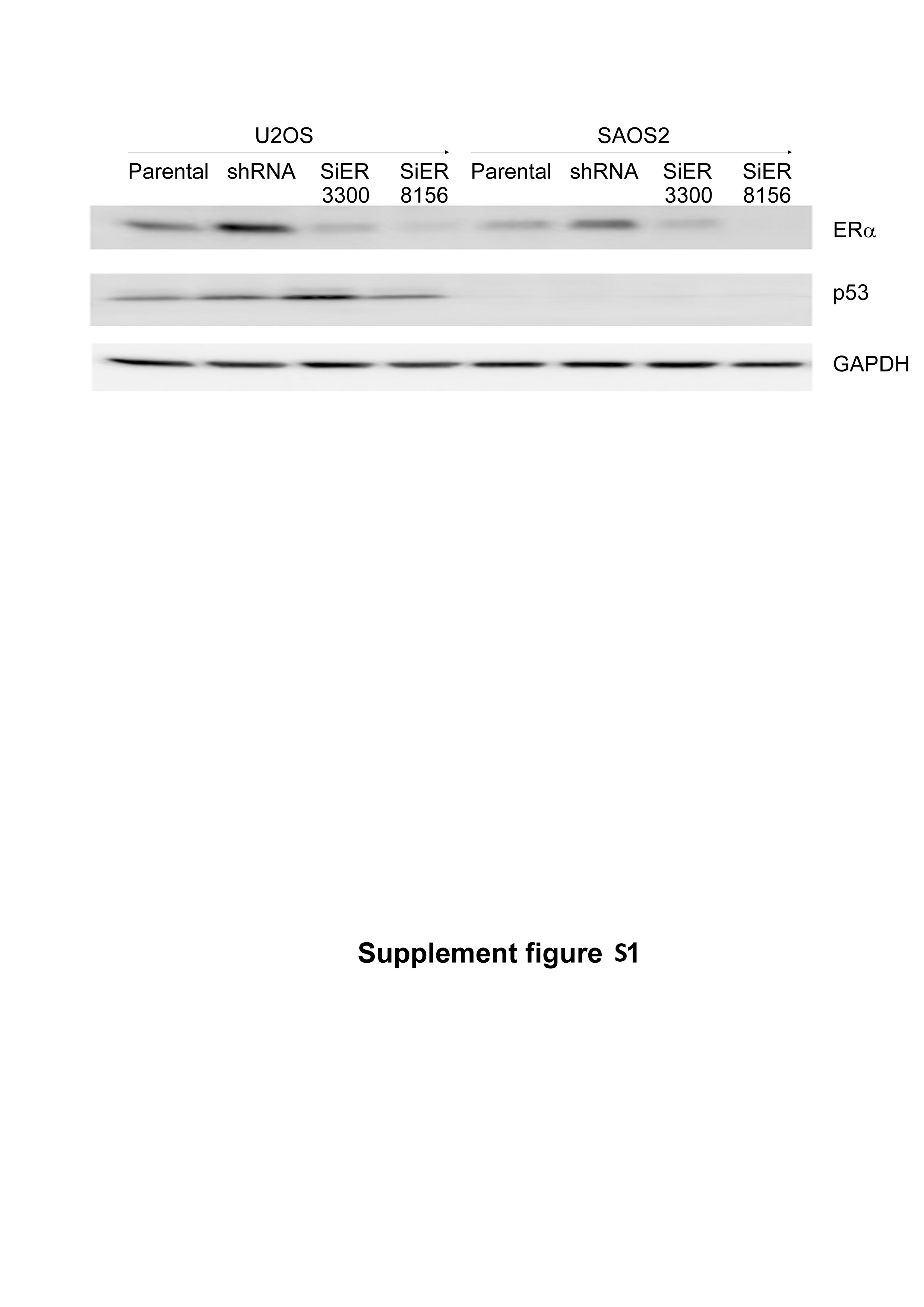

Supplement: Supplementary file 1 [file ijms-22-11238-s001.zip › Supplement figure 1-IJMS.tif]

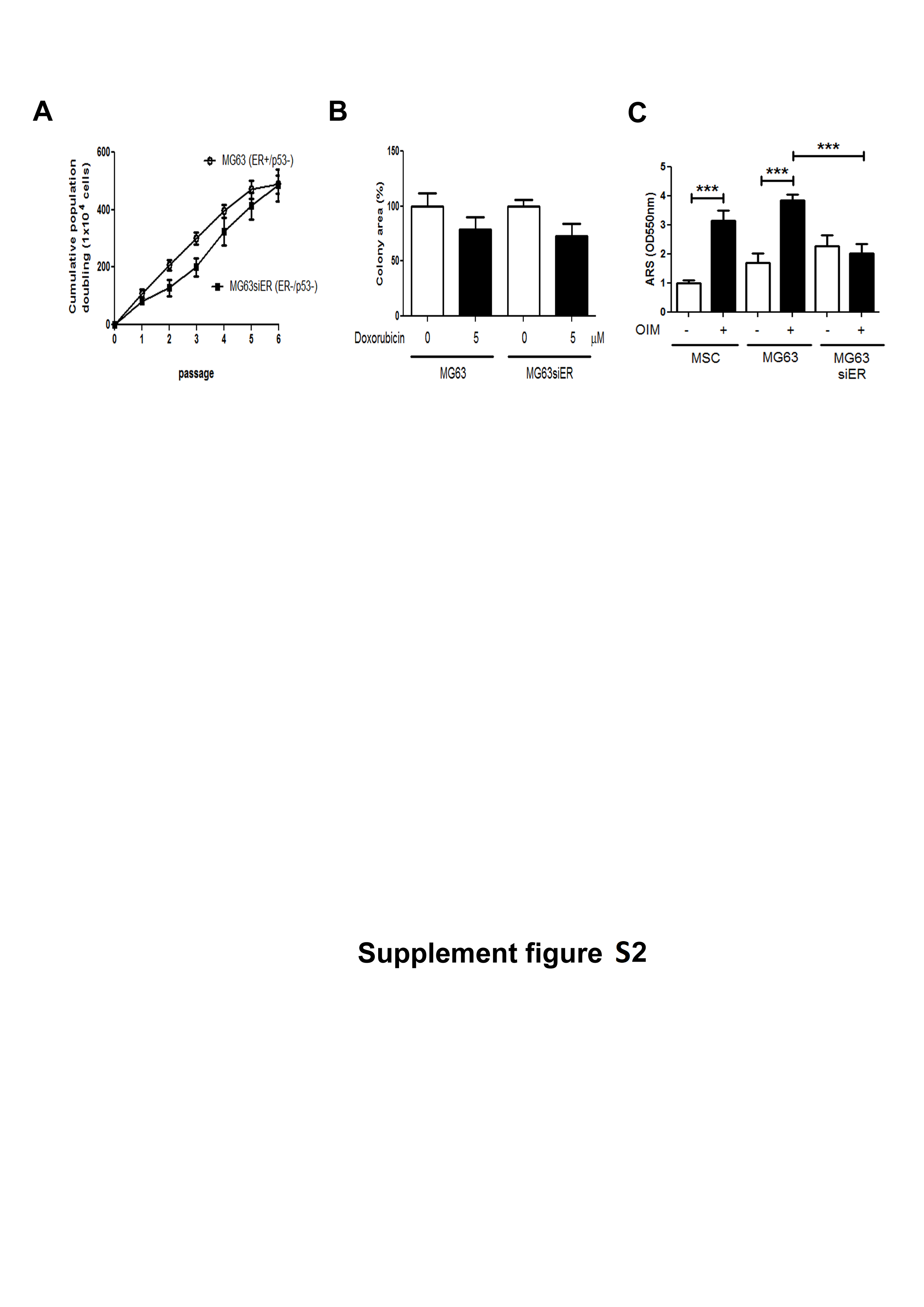

Supplement: Supplementary file 1 [file ijms-22-11238-s001.zip › Supplement figure 2-IJMS.tif]

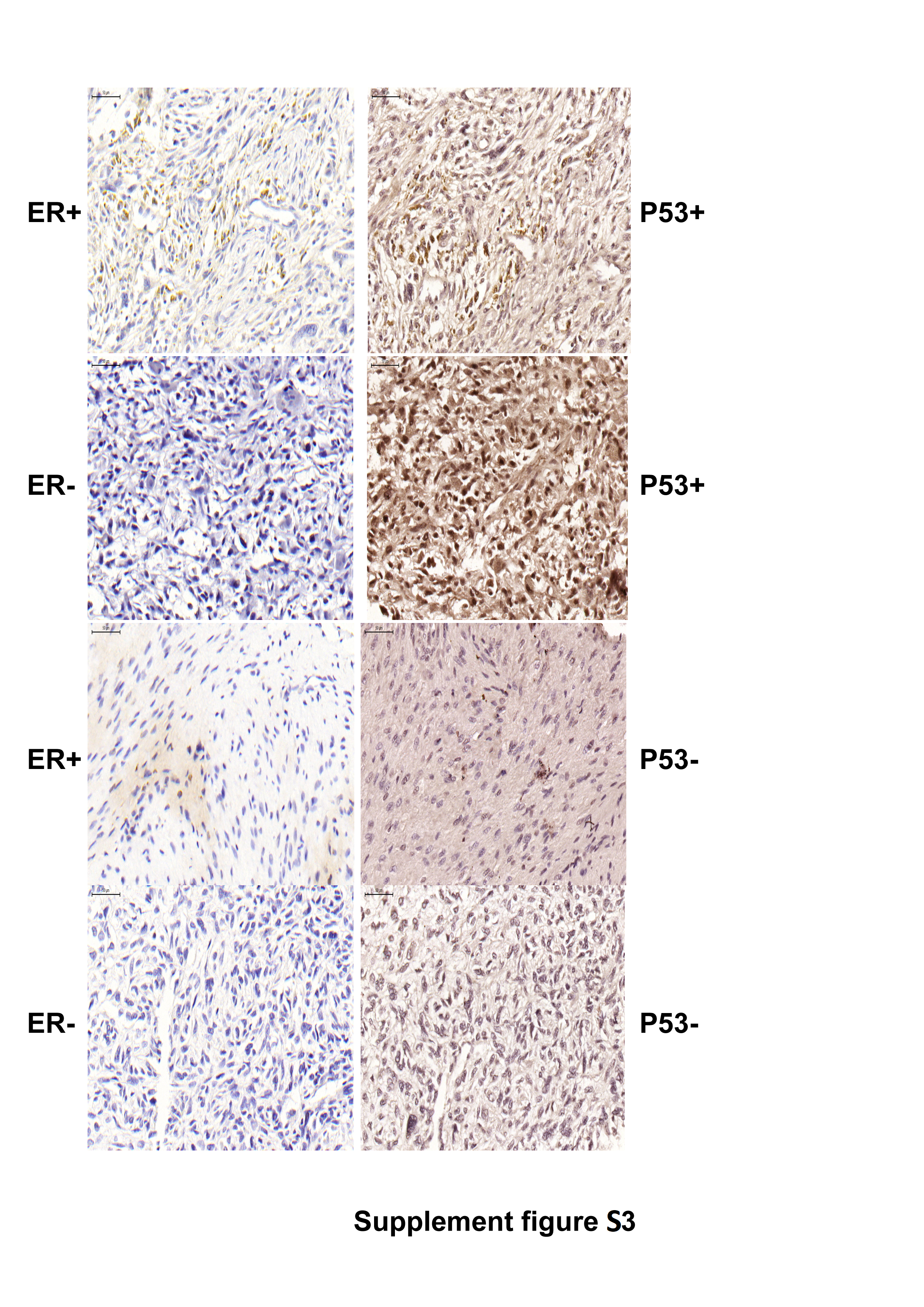

Supplement: Supplementary file 1 [file ijms-22-11238-s001.zip › Supplement figure 3-IJMS.tif]
